# Molecular Processes in Biological Thermosensation

**DOI:** 10.1155/2008/602870

**Published:** 2008-05-12

**Authors:** I. Digel, P. Kayser, G. M. Artmann

**Affiliations:** Laboratory of Cellular Biophysics, Aachen University of Applied Sciences, Ginsterweg 1, 52428 Juelich, Germany

## Abstract

Since thermal gradients are almost everywhere, thermosensation could represent one of the oldest sensory transduction processes that evolved in organisms. There are many examples of temperature changes affecting the physiology of living cells. Almost all classes of biological macromolecules in a cell (nucleic acids, lipids, proteins) can present a target of the temperature-related stimuli. This review discusses some features of different classes of temperature-sensing molecules as well as molecular and biological processes that involve thermosensation. Biochemical, structural, and thermodynamic approaches are applied in the paper to organize the existing knowledge on molecular mechanisms of thermosensation. Special attention is paid to the fact that thermosensitive function cannot be assigned to any particular functional group or spatial structure but is rather of universal nature. For instance, the complex of thermodynamic, structural, and functional features of hemoglobin family proteins suggests their possible accessory role as “molecular thermometers”.

## 1. Introduction

Temperature changes are one of the main stresses experienced by organisms from bacteria to plants and animals and therefore temperature is one of the environmental cues under constant vigilance in living cells. Several problems arise from exposing a cell to a sudden change in temperature [1]: firstly, membrane fluidity changes, that affect many membrane-associated vital functions. Secondly, nucleic acid topology will be affected causing shifts in processes such as transcription and translation. Finally, the protein function is affected both from structural and catalytic points.

Hence, living cells need devices for sensing environmental temperature changes in order to adapt their biochemical processes accordingly. A successful adaptive response to temperature changes cannot be performed by corresponding changes in the rate and equilibrium of enzymatic reactions only. Such a mechanism of adaptive reaction is too unspecific and uncontrollable. To cope with temperature variation, living organisms need sensing temperature alterations and translating this sensory event into a pragmatic gene response.

While such regulatory cascades may ultimately be complicated, it appears that they contain primary sensor machinery at the top of the cascade. The functional core of such machinery is usually that of a temperature-induced conformational or physicochemical change in the central constituents of the cell. Hence, a specific sensory transduction mechanism is needed, including, as a key element, a molecular sensor, transforming physical parameter (temperature) into a biologically significant signal (change in membrane permeability, specific inhibition/stimulation of gene expression, etc.). In a sense, a living organism can use structural alterations in its biomolecules as the primary thermometers or thermostats. Thus, sensory transduction is a complex biological process aimed at integrating and decoding physical and chemical stimuli performed by primary sensory molecular devices. Furthermore, sensory perception of potentially harmful stimuli functions as a warning mechanism to avert potential tissue/organ damage.

Among temperature-controlled processes in living organisms, most well-known are the expression of heat-shock and cold-shock genes [2]. Relocation of a culture of *Escherichia coli* adapted to an optimal growth to a sudden temperature increase, or decrease, by some 10–15°C results in adaptive shock responses. Such responses involve a remodeling of bacterial gene expression, aimed at adjusting bacterial cell physiology to the new environmental demands [3, 4]. The response of prokaryotic and eukaryotic systems to heat-shock stress has been investigated widely in a large number of organisms and model cell systems. Notably, all organisms from prokaryotes to plants and higher eukaryotes respond to cold and heat shock in a comparatively similar manner. The general response of cells to temperature stress (cold or heat) is the elite and rapid overexpression of small groups of proteins, the so-called CSPs (cold-shock proteins) or HSPs (heat shock proteins), respectively, but the initial launching mechanism is different in both cases.

In bacteria, the heat response generally invokes some 20 heat-shock proteins, whose functions are primarily to help deal with, and alleviate, the cellular stress imposed by heat [5]. Many of these proteins participate in reconstituting and stabilizing protein structures and in removing misfolded ones. The expression of this special chaperone system, which includes the proteins *DnaK*, *DnaJ,* and *GrpE* is activated by the presence of misfolded, temperature-denatured proteins. Thus, one could implicate the binding of partially unfolded proteins by chaperones as the thermosensoric event regulating expression of heat-shock proteins, where the primary sensory element is constituted by some easily denaturing proteins. This, in turn, demonstrates that even bacteria can practically utilize destructive changes in protein conformation as a means for temperature sensing.

In case of cold shock, the primary sensing event is more obscure. Various reports have now shown that when in vitro cultivation temperature is lowered, the rigidity of the cell membrane is increased which results in compromised membrane-associated cellular functions. Furthermore, cold stress dramatically hinders membrane-bound enzymes, slows down diffusion rates, and induces cluster formation of integral membranous proteins [6].

In mammalian cells the five known mechanisms by which cold-shock-induced changes occur in gene expression are: (i) a general reduction in transcription and translation, (ii) inhibition of RNA degradation, (iii) increased transcription of specific target genes via elements in the promoter region of such genes, (iv) alternative pre-mRNA splicing, and (v) via the presence of cold-shock specific IRESs (internal ribosome entry segments) in mRNAs that result in the preferential and enhanced translation of such mRNAs upon cold shock [7].

It has been pointed out that cold stress exposes cells to two major stresses: those relating to changes in temperature and those related to changes in dissolved oxygen concentration at decreased temperature, and it is therefore necessary to consider potential responses to each, either independently or as part of a coordinated response. Separating the relative effects of temperature and oxygen as a result of decreased temperature is difficult and has not been extensively addressed to date. Both changes in dissolved oxygen and temperature reduction result in similar changes in cultured mammalian cells [7].

The shock response systems discussed above belong to ultimate mechanisms aimed to survival under extreme temperature conditions. However, the ability to express certain factors can be affected by reasonably small temperature changes. Less drastic changes in temperature may not induce shock responses, but can be sufficient to modulate the expression of virulence genes, for example in *Shigellae* [8] and *Yersiniae* [9]. While one might be surprised that organisms built on such minimalist approaches as bacteria respond to temperature changes, the consequence of these observations is that even bacteria actually sense temperature shifts in order to control gene expression accordingly. Investigators have now been studying the moderate temperature sensation in a variety of organisms for at least several decades or more. Recently, a number of reports have shown that exposing yeast or mammalian cells to sub-physiological temperatures (<30°C or <37°C, resp.) invokes a coordinated cellular response involving modulation of transcription, translation, metabolism, the cell cycle and the cell cytoskeleton [7, 10–13]. Nevertheless very little is known about the molecular mechanisms that govern initial response on small thermal stimuli, particularly the primary sensory transduction mechanisms.

Below, we have tried to uncover some aspects of the molecular basis of temperature sensing by biological molecular thermometers, to summarize some known aspects of primary components of temperature signal transduction and to show possible thermosensitive role of even “common” molecules such as hemoglobin.

## 2. Temperature-Sensing Biomolecules

In addition to specificity and sensitivity, the pragmatic thermoresponse should be one that is reversible and controlled. Such complexity of thermosensing and thermoregulation may reflect the demands to handle and fine-tune responses to an important environmental factor in a dynamic fashion. However, ultimately, it seems that basic and uncomplicated biochemical processes are used as primary sensors and, for that purpose changes in the nucleic acid, protein or membrane physicochemical state appear highly suitable. Bellow we make a short overview of temperature-sensing properties of most important groups of biological macromolecules.

### 2.1. Membrane Lipids

While the information available is somewhat scant, the picture emerging shows that cells can use signals generated through changes in nucleic acid or protein conformation, or changes in membrane lipid behavior, as sensory devices. The physical state of membranes does change in response to temperature shifts in phase-transition manner [14], but the temperature-induced changes in real biological membranes are not sharp because many kinds of fatty acids present, having different characteristic temperature points of phase transition. Thus, it would not be surprising if cells (even those of bacteria) could utilize, changes in membrane fluidity as a thermometer device, assisted by protein helpers, playing a role of switchers, “sharpening” the temperature response. Microorganisms counteract the propensity for membranes to rigidify at lower temperature by adapting to the conditions in order to maintain a more-or-less constant degree of membrane fluidity (homeoviscous adaptation). The cyanobacterium *Synecocystis* responds to decreased temperature by increasing the cisunsaturation of membrane-lipid fatty acids through expressing acyl-lipid desaturases [15–17]. Lipid unsaturation would then restore membrane fluidity at the lower temperature. In *B. subtilis*, this lipid modification is initiated through the activity of a so-called two-component regulatory system consisting of the DesK and DesR proteins [15]. Prokaryotic two-component regulatory systems usually consist of protein pairs, a sensor kinase and a regulatory protein [18].

It appears that it is a combination of membrane physical state and protein conformation that is able to sense temperature and to translate this sensing event into proper gene expression. However, sensing of temperature through alteration in nucleic acid conformation could be more efficient temperature-mediated mechanism of gene expression.

### 2.2. RNA

Messenger RNAs apart from carrying their coding information for protein generation are also rapidly emerging as regulators of expression of the encoded message. With unique chemical and structural properties, sensory RNAs perform vital regulatory roles in gene expression by detecting changes in the cellular environment through interactions with small ligands [19, 20] and proteins [21, 22].

Regulatory RNA elements, “riboswitches,” have been reported recently, responding to intracellular signals by conformational changes. Riboswitches are conceptually divided into two parts: an *aptamer* and an expression platform. The aptamer directly binds the small molecule, and the expression platform undergoes structural changes in response to the changes in the aptamer. The expression platform is what regulates gene expression. Riboswitches demonstrate that naturally occurring RNA can specifically response on versatile physical and chemical stimuli, a capability that many previously believed was the domain of proteins or artificially constructed RNAs called aptamers [23].

Theoretically, RNA molecules have a strong potential as temperature sensors, in that they can form pronounced secondary and tertiary structures [24], and through their ability to form intermolecular RNA : RNA hybrids [25]. Both of these processes greatly depend on the formation of complementary base pairing, and consequently one would anticipate these to be dependent on environmental temperature.

RNA thermometers operate at the post-transcriptional level to sense selectively the temperature and transduce a signal to the translation machinery via a conformational change. They have usually a highly structured 5'-end that shields the ribosome binding site at physiological temperatures [1, 26–29]. Changes in temperature are manifested by the liberation of the Shine-Dalgarno (SD) sequence, thereby facilitating ribosome binding and translation initiation.

### 2.3. DNA

It is known that both in prokaryotic and eukaryotic cells, the geometry and tension of DNA are highly dynamic and correspond to its functional activity. In the bacterial cell, chromosome and plasmid DNA is contained in a “twisted” superhelical conformation [30, 31], where the degree of superhelicity varies in response to changes in the ambient temperature. In many examples, the expression of many genes is dependent on DNA conformation, and temperature-dependent gene regulation is mastered through changes in DNA supercoiling [3, 32, 33].

Seemingly, the temperature-induced conformational changes in DNA are mainly controlled through the presence of “nucleotid-associated” proteins, of which H-NS is the best characterized [30, 34]. In *E. coli*, creating and maintaining conformational structures in the DNA molecule are mainly regulated through the balance of two opposing topoisomerase activities, mainly those of topoisomerases II and I [35, 36].

Examples of pure DNA-related temperature sensitivity are rare if ever reported. In most cases, genomic thermosensitivity appears to be a result of certain interplay among DNA, RNA, and proteins. Some bacteria carry a DNA-plasmid which shows a controlled constant plasmid copy number at one temperature and a much higher or totally uncontrolled copy number at a different temperature. The high-copy number phenotype of pLO88 plasmid maintained in *Escherichia coli* (HB101) is observed only at elevated temperatures, (above 37°C), and is due to the precise position of a Tn5 insertion in DNA, but the exact mechanism remains obscure [37].

All abovementioned examples of membrane- and nucleic acid-based temperature sensitivity apparently include proteins as a key regulatory component. Therefore, from the point of view of molecular temperature sensation, protein-based molecular “thermometers” represent an extremely interesting group.

### 2.4. Proteins

Many sensory pathways in living organisms use structural changes in proteins as a primary perceptive event, activating further signaling cascades. If *E. coli* is exposed to an oxidative substance such as hydrogen peroxide, it responds by the activation of a transcriptional regulator protein OxyR [38]. Activation of OxyR is achieved through the formation of a disulphide bound within the protein, upon which OxyR induces the expression of a set of genes adapting the bacterial cell to oxidative stress. This illustrates how it is possible both to *sense* and respond to an abrupt change in a specific environmental factor in a simple, yet elegant mode.

One would expect the organisms and cells to be similarly elegant when sensing temperature shifts. Indeed, a striking example is the temperature-controlled switching of the flagellar rotary motor of *E. coli* between the two rotational states, clockwise (CW) and counterclockwise (CCW) [39]. The molecular mechanism for switching remains unknown, but seems to be connected to the response regulator CheY-P. Two possible models of CheY-P action explain shifting the difference in free energy between CW and CCW states in terms of (i) conformation-related differential binding [40, 41] and (ii) thermodynamic changes in dissociation constants [42].

Further studies on the thermosensory transducing system in *E. coli* revealed that two major chemoreceptors, *Tar* and *Tsr*, which detect aspartate and serine, respectively, also function as thermoreceptors, as well as *Trg* and *Tap* receptors [43]. Interestingly, in spite of different specificity and sensitivity, amino acid sequences of all four chemoreceptors have a significant homology. These are transmembrane proteins with two functional domains in their role as chemoreceptors; one is a ligand-binding domain located in the periplasm and the other is a signaling domain located in the cytoplasm. Thus, it is suggested that a temperature change induces a conformational change in these two receptors and that this conformational change triggers the signaling for thermoresponse. In the simplest model of thermoreception by these receptors, two conformational states of these receptors are assumed: a low-temperature state and a high-temperature state [44]. The swimming pattern of the *Trg*- and *Tap*-containing cells was determined simply by the temperature of the medium, indicating that these cells under nonadaptive conditions *sense the absolute temperature as the thermal stimulus*, *and not the relative change in temperature*.

The understanding of proteins temperature-related sensory transductions in terms of their underlying molecular mechanism is fast-advancing thanks to the discovery and functional characterization of the transient receptor potential (TRP) channels. This protein family, first identified in *Drosophila*, is at the forefront of our sensory stem, responding to both physical and chemical stimuli and, thus, having diverse functions [45, 46].

The superfamily of TRP channels currently comprises nearly 30 mammalian members grouped into six related families: TRPC, TRPV, TRPP, TRPM, TRPN, and mucolipins. In higher organisms, TRPV channels are important polymodal integrators of noxious stimuli mediating thermosensation and nociception. The transient receptor potential channel vanilloid receptor subunit 1 (TRPV1) is widely recognized as a molecular integrator of physical and chemical stimuli in the peripheral nociceptor terminals [11, 47].

A subset of these channels, the thermo-TRPs, is activated by distinct physiological temperatures. Six thermo-TRP channels, which are all characterized by their unusually high-temperature sensitivity (*Q*_10_ > 10), have been cloned: TRPV(1)–(4) are heat-activated [48–50], whereas TRPM8 [50, 51] and TRPA1 [52] are activated by cold. With a *Q*_10_ of about 26 for TRPV1 [53] and approx. 24 for TRPM8 [54, 55], they far surpass the temperature dependence of the gating processes characterized by other ion channels (*Q*_10_ > 3) [53]. In spite of the great advances made, the molecular basis for regulation by temperature remains unknown because of the lack of structural information. More detailed consideration of protein dynamics and thermodynamics can bring us closer to understanding of universal principles of thermal sensation.

## 3. Biophysical Aspects of Protein-Aided Thermosensation

It appears from the above mentioned examples of protein participation in temperature sensing events that sudden conformational changes, “structural transitions” play essential role on the primary conversion of physical stimulus into biologically relevant signal.

Phase transitions and critical phenomena continue to be the subject of intensive experimental and theoretical investigation. In this context, systems consisting primarily of well characterized proteins and water can serve as particularly valuable objects of study. The importance of studies of specific phase transitions in protein/water solutions derives also from their physiological relevance to the supramolecular organization of normal tissues and to certain pathological states. For example, such phase transitions play an important role in the deformation of the erythrocyte in sickle-cell disease [21, 56] and in the cryoprecipitation of immunoglobulins in cryoglobulinemia and rheumatoid arthritis [57].

Discussions about protein stability and temperature-induced structural transitions are usually limited to the stability of the native state against denaturation. Yet the native state may include different functionally relevant conformations characterized by different Gibbs energies and therefore different stabilities (e.g., the R and T states of hemoglobin). Even when the native state does not undergo a conformational change, it is still characterized by the occurrence of a large number of local unfolding events that give rise to many substates. Thus, the native state itself needs to be considered as a statistical ensemble of conformations rather than unique entity. These distinctions are very important from the functional point of view since different conformations are usually characterized by different functional properties.

The stabilizing contributions that arise from the hydrophobic effect and hydrogen bonding are largely offset by the destabilizing configurational entropy. The hydrophobic effect is strongly temperature-dependent, and is considerably weaker and perhaps even destabilizing at low temperatures than at elevated temperatures. The contribution of various interactions for a “typical” protein is reported in many works [58–62]. Apparently, the transition from stabilizing to destabilizing conditions is achieved by relatively small changes in the environment. These can be changes in temperature, pH, and addition of substrates or stabilizing cosolvents. While the exact contribution of different interactions to the stability of globular proteins remains a question, our understanding seems to be refined enough to allow for the reasonable prediction of the overall folding thermodynamics [61, 62]. Important to mention that both the enthalpy end entropy changes are not constant but increasing functions of temperature, and that the Gibbs energy stabilization of a protein can be written as follows:

(1) $$\Delta G=\Delta H(T_{R})+\Delta C_{p}(T-T_{R})-T\Delta S(T_{R})+\Delta C_{p}\ln\;(T/T_{R}),$$

where *T*_*R*_ is a convenient reference temperature. Δ*C*_*p*_ is the heat capacity change, and Δ*H*(*T*_*R*_) and Δ*S*(*T*_*R*_) are the enthalpy and entropy values at that temperature. The temperature dependency of Δ*H* and Δ*S* is an important issue because it transforms the Gibbs energy function from a linear into a parabolic function of temperature.

For large values of Δ*C*_*p*_, the Gibbs Energy crosses zero point twice—temperature (heat denaturation) and one at low temperature (cold denaturation). The native state is thermodynamically stable between those two temperatures and Δ*G* exhibits a maximum at the temperature at which Δ*S* = 0. The peculiar shape of the Gibbs energy function of a protein does not permit a unique definition of protein stability. For example, having a higher denaturation temperature does not necessarily imply that a protein will be more stable at room temperature. Within the context of the structural parameterization of the energetics, the Gibbs energy of protein stabilization is approximated by

(2) $$\Delta G=\Delta G_{\text{gen}}+\Delta G_{\text{ion}}+\Delta G_{\text{tr}}+\Delta G_{\text{other}},$$

where Δ*G*_gen_ contains the contributions typically associated with the formation of secondary and tertiary structure (van der Waals interactions, hydrogen bonding, hydration, and conformational entropy), Δ*G*_ion_ the electrostatic and ionization effects, and Δ*G*_tr_ the contribution of the change in translational degrees of freedom existing in oligomeric proteins. The term Δ*G*_other_ includes interactions unique to specific proteins that cannot be classified in a general way (e.g., prosthetic groups, metals, and ligands) and must be treated on a case-by-case basis.

Nilius and coworkers have recently applied a simple thermodynamic formalism to describe the shifts in voltage dependence due to changes in temperature [63, 64], where the probability of the opening of a protein channel is given as a function of temperature, the gating charge, Faraday's constant, and the free-energy difference between open and closed states of the channel.

At biological temperatures, some proteins alternate between well-defined, distinct conformations. In order for two conformational states to be distinct, there must be a free-energy barrier separating them. The notions involved to get from one state to another are usually much more complex than the oscillation of atoms and groups about their average positions. In proteins, because most of the forces that stabilize the native state are noncovalent, there is enough thermal energy at physiological temperature for weak interactions to break and reform frequently. Thus a protein molecule is more flexible than a molecule in which only covalent forces dictate the structure.

To further understand the nature of dynamic transitions in proteins, it is particularly important to characterize solvent effects. Solvent can in principle affect protein dynamics by modifying the effective potential surface of the protein and/or by frictional damping. Changes in the structure and internal dynamics of proteins as a function of solvent conditions at physiological temperatures have been found by using several experimental techniques [65]. It is clear from the works of Zaccai and others that solvent affects protein dynamics at physiological temperatures [66–68]. They reported that in the absence of minimal hydration, proteins do not function at all. Therefore, a solvent dependence of the dynamic transition might be expected. Indeed, measurements on CO binding to myoglobin indicate that dynamic behavior of the protein is correlated with a glass transition in the surrounding solvent [69], and a recent molecular dynamics analysis of hydrated myoglobin also indicates a major solvent role in protein dynamic transition behavior [70].

From the point of view of structural biophysics, thermosensation is a special sort of mechanosensation and therefore many theoretical models and considerations developed for protein mechanosensors are also applicable for thermosensors. The difference between mechanosensitive channels and thermosensitive molecules is only the size and the organization of “pushing” agents—a lot of noncoordinated events (thermal stimuli) versus a net stretch (mechanical stimuli). Interestingly, many members of thermosensing TRPV family are known osmo- and mechanosensors. Because mechanical stimuli are everywhere, mechanosensation could represent one of the oldest sensory transduction processes that evolved in living organisms. Similar to thermal sensors, what exactly makes these channels respond to membrane tension is unclear. The answer will not be simple, because not thermal and mechanosensors are very diverse [71, 72]. However, there are interesting parallels in structural composition of different classes of known temperature-sensory proteins.

## 4. Structural Features of Protein Molecular Thermometers

Despite significant evolutionary distances and apparent differences of primary structure all temperature-sensitive proteins known so far display some remarkable similarities in their tertiary/quaternary structure. The ability of a big protein TlpA responsible in *Salmonella typhimurium* for temperature regulation of transcription resides in its structural design. Two-thirds of the C-terminal portion of TlpA is contained in an alpha-helical-coiled-coil structure that constitutes an oligomerization domain. As the temperature increases, the proportion of DNA-binding oligomers decreases, leading to a derepression of the target gene. At moderate temperatures, the concentration of TlpA increases, shifting the balance to the formation of DNA-binding oligomers and, in part, restoring the repression potential of TlpA. Thus, TlpA undergoes a reversible conformational shift in response to temperature alteration, leading to an alteration in the oligomeric structure and subsequently in the regulatory capacity of TlpA [44].

The sensory capacity is contained in the coiled-coil structure of TlpA, which illustrates the means of sensing temperature through changes in protein conformation. The coiled-coil structure is a versatile and a rather flexible motif in mediating protein: protein interactions. In vertebrates, the thermosensitive elements of transcriptional mechanism typically contain coiled-coil folding motifs, such as those in leucine zipper family.

TRPV channel subunits in turn have a common topology of six transmembrane segments (S1–S6) with a pore region between the fifth and sixth segments, and cytoplasmic N- and C-termini. In both TRPV1 and TRPM8, modulation of channel gating behavior by temperature arises from the C-terminal structure that follows the S6 inner helix [51]. Partial deletions performed in the C-terminal domain of TRPV1 result in functional channels with attenuated heat sensitivity, and truncation of the whole TRPV1 C-terminal domain completely hindered channel expression [53]. Interestingly, in TRPM8 channels, binding of phosphatidylinositol bisphosphate (PIP2) leads to channel activation [73]. The proximal C-terminal TRP domain is conserved in TRPM8 and appears to serve as a PIP2 site [74]. These observations, and the fact that the key question regarding what makes thermo-TRPs temperature sensitive remained unanswered, suggests building C-terminal chimeras between different members of TRPV family as a further step in structural approach [11].

In thermo-TRP channels, it has been proposed that the structural rearrangement leads to a change in tension on the helical linker connecting the C-terminal domains with S6 segment. This tension on the linker provides the energy necessary to move the S6 inner helix to the open conformation [54, 55]. Another possibility could be that temperature affects the interaction between a particular portion of the proximal C-terminal and some other region of the channel, probably an intracellular loop. Finally, it may be that independent arrangements induced by temperature on C-terminal domains directly promote gate opening [53].

Bernd Nilius' group in their study on the voltage dependence of TRP channel gating by temperature pointed out that the small gating charge of TRP channels compared to that of classical voltage-gated channels could lie at the basis of the large shifts of their voltage-dependent activation curves, and may be essential for their gating versatility [63, 64]. Thus, small changes of the free energy of activation of these channels can result in large shifts of their voltage-dependent activation curves, and concomitant gating of these channels.

In membrane, TRP channels form tetramers of identical subunits [47]. The crystal structure of mechanosensitive/thermosensitive membrane proteins reveals that the channel folds as a homoheptamer that has a large, cytoplasmic region. Recently obtained data indicate that the modular nature of the structures involved in activation processes allow different stimuli (voltage, temperature, and agonists) to promote thermo-TRP channel opening by different interrelated mechanisms as has been suggested in the form of allosteric interaction [54, 55, 75].

The very interesting aspect resides in the observation that bacterial proteins H–NS and StpA may form hetero-oligomers exactly the same way as TRPV thermosensory channels of higher animals sometimes do [30, 54]. In this context, it is important to note that the temperature-sensitive H–NS function is also associated with oligomerization and that the H–NS oligomerization domain most evidently relies on the formation of coiled-coil oligomers [31, 69].

The molecular dynamics and organization of the temperature-sensing proteins signaling complexes are still elusive, although fast-advancing progress in this arena is uncovering the molecular identity of these elements. A series of papers published by Artmann and coworkers revealed intriguing temperature-related structural transitions phenomena in hemoglobins (Hb) and myoglobins of different species [58, 76, 77]. The reported nonlinearity in hemoglobin temperature behavior seems to be connected to physiological body temperature of the given species and therefore might surprisingly reflect the role of Hb as a molecular thermometer [78].

## 5. Novel Classes of Molecular Thermometers: Hemoglobin and CO

Proteins of the hemoglobin (Hb) family, also referred to as the myoglobin (Mb) or globin family are gas-binding heme proteins found in all domains of life. Hbs have evolved slightly different structures and functions, but both the predominantly helical structure and certain aminoacids are well conserved (1). Distinct-but-related classes of Hbs are widespread in Bacteria, Archaea, and Eucarya. Although the physiological functions of vertebrate Hbs known so far are the transport of molecular O2 and have a role in nitric oxide (NO) metabolism, those of nonvertebrate Hbs are much more diverse. In addition to O2 transport and storage, they include facilitation of O2 diffusion, reactions with sulfide and its transport, complex and as yet incompletely elucidated roles in NO regulation and metabolism, maintenance of acid-base balance, O2 scavenging, O2 sensing, oxidase and peroxidase activities, the latter related to detoxification, vitellogenin-like function and roles as light-shading pigments and regulators of the buoyancy of aquatic insects [79].

The reported [58, 76, 77] temperature effects on hemoglobin hydration and aggregation may reflect an unknown, possibly atavistic, yet expectable function of Hb in keeping homeostasis.

The suggested by Zerlin et al. [78] ability of mammalian hemoglobins to thermoadaptation finds support in many studies made for thermophilic organisms. A gene encoding a protein homologous to Hb was identified in *Aquifex aeolicus*, a hydrogen-oxidizing obligate chemolithoautotroph that grows at temperatures of >95°C under microaerobic conditions. *A. aeolicus* thermoglobin, AaTgb, is monomeric, resistant to thermal and chemical denaturation, pentacoordinate in the ferrous deoxygenated state, and oxygen-avid. Key strongly, although not strictly, conserved positions are preserved in the AaTgb sequence. Proline occupies the C2 position, initiating the start of the C helix. Although histidine occupies the distal E7 position in most plant and animal Hbs, this residue is commonly adaptively replaced by glutamine in many invertebrate and bacterial Hbs. Similarly large thermal variations are also encountered by Hb-containing prokaryotes like the cyanobacterium *Nostoc* that extends from tropical to polar terrestrial environments [79]. Most of thermophilic hemoglobins discovered so far may be described as basic ones. The aminoacid sequence is compact, without additional residues or domains at either terminus beyond the A and H helices of the canonical fold. This basic fold may even be fused to other domains or duplicated and fused onto itself to yield Hbs with multiple copies of the globin domain.

The equilibrium constants for dimer-tetramer association of Hb have been determined as a linear function of temperature from kinetic studies of the forward and reverse rate constants [60]. It is worthy to note that these studies have been performed at temperatures below 30°C and therefore do not correspond to physiological conditions. The thermodynamic parameters calculated for Hb are consistent with an increased role of hydrophobic interactions within the dimer-dimer contact region, or a decreased role of hydrogen bonds and ion pair interactions.

Thermodynamic experiments by Frauenfelder, Petsko, and Tsernoglu [80] showed that myoglobin can assume a large number of slightly different structures, conformational substates, separated by energy barriers. Evidence for multiple potential energy minima also comes from molecular dynamics simulations made for myoglobin. The complex of thermodynamic, structural, and functional features of hemoglobin family proteins supports the hypothesis of their possible secondary role as temperature-sensing molecules. For homeothermic organisms (birds and mammals) such multiple protein-mediated temperature control could be of special importance, supporting its strengthening during evolution.
